# Primary Health Care. Specific Nursing Leadership*

**DOI:** 10.17533/udea.iee.v40n3e16

**Published:** 2023-02-14

**Authors:** José Ramón Martínez-Riera

**Affiliations:** 1 Nurse, Full professor, Universidad de Alicante; Spain. President Community Nursing Association (AEC, for the term in Spanish). Email: jr.martinez@ua.es Universidad de Alicante Universidad de Alicante Spain jr.martinez@ua.es

The 1978 Alma-Ata International Declaration (Kazakhstan - former Soviet Socialist state),(1) was a turning point, at least regarding the conception of what was understood by primary health care (PHC) and what it should contribute to respond to the needs and demands of the population. The Declaration considered it, not only possible, but was aimed at achieving health for every country by the year 2000. The approaches to achieve this, although praiseworthy and difficult for anyone to reject, led to its signing by the 134 countries and 67 international organizations present at the meeting - highlighting the important absence of the People’s Republic of China-, the intention was to stop financing rearmament policies and support for military conflicts and direct policies to promote independence, peace, *détente* and disarmament, to use those resources in the social and economic development of the people and invest on PHC, as essential element to achieve it.

But beyond these proposals with an eminently political nature and always subject to the interests and developments of social, economic, and demographic events… it is important to highlight something that unfortunately has received enough importance and visibility, such as the express request for health professionals to commit to promoting PHC in every corner of the planet. Something that was to be key in the evolution of PHC, although with unequal enthusiasm, involvement, and commitment by the main professionals from the different disciplines that made up the PHC staff.

The years after the Alma-Ata Declaration brought about an infinity of reactions from the member states, health professionals, the pharmaceutical industry, trade unions, and the different organizations representing citizens and users of the health systems, so that initiating and developing the strategy proposed in the Declaration was a path strewn with obstacles. Voices of detractors and supporters emerged. The positioning arguments of the different institutions that, with a progressive vision, defended the strategy focused the discourse and little by little a timid but continuous impulse was implanted in the countries that lacked it and where it was necessary to refocus primary medical care, for the implementation of PHC as the population's first contact with the National Health System.

The 1980s saw the establishment of a conservative hegemony initiated by Ronald Reagan and Margaret Thatcher, which quickly triumphed and is still largely in force today. Such was based on two objectives: general privatization and the triumph of social individualism. This resulted in the state’s abandonment of social responsibilities and disappearance of the concept of "social" as the right of people to a fair distribution of wealth. Let us recall, herein, the famous quote by the "Iron Lady": *There’s no such thing as society, there are only individual men and women and families*.(2)

After 20 years since the enactment of the Declaration and for the purpose of evaluating the objectives proposed in it for the year 2000, the international community, during the Fifty-first World Health Assembly held in May 1998, adopted a new Global Health Declaration in which its members, through five articles, recognize *“that the enjoyment of the highest attainable standard of health for a human being is a fundamental right; that they join the ethical principles of equity, solidarity, and social justice”.* (3) For this, they reformulate the initial objective of the Alma-Ata Declaration, moving on to proposing the “Health for All (HFA) in the 21st century” through pertinent regional and national policies and strategies and committing to strengthening, adapting, and reforming the sanitary health systems, ensuring the essential PHC elements, adopting for such a new commitment by the undersigning countries.

After 40 years of the Alma-Ata Declaration, a new declaration is signed, the Astana Declaration,(4) that charts the path to achieve universal health coverage. In October 2018, the meeting took place in Astana (Kazakhstan) under the auspices of the World Conference on Primary Health Care, Heads of State and Government and the representative ministers, to evaluate the evolution of World Health from the Alma-Ata World Conference on Primary Health Care. In his statement, the General Director of the World Health Organization, Tedros Adhanom Ghebreyesus, said that *“Today, instead of Health for All, we have health for some”*, while proclaiming that *“We all have a solemn responsibility to ensure that today's Declaration on primary health care enables everyone, everywhere, to exercise their fundamental right to health.”*

This perception by the General Director could be subscribed by all the professionals who work or have worked in PHC in any country over the years PHC has been developed, who have worked convinced that the goals proposed could be reached. But over time, they have been witnesses and partly protagonists of the deterioration of the health reform process where Primary Care should be the “gateway to the system” and receive the resources and political effort necessary for it to be so. Aware of that deterioration, they have struggled against the winds blowing at any given moment to avoid this as much as possible and to maintain their commitment to the community to which they provide care.(5)

Thus, what began as an important component of an illusionary discourse in the 1980s was diluted over the years to give way to a panorama that in no way resembled what was touted. Disillusionment and demotivation contributed to the routine of daily care activities and tasks occupying the gaps left by the abandonment of initiatives and strategies that during the initial years of the denominated reform had much relevance, at least rhetorical, like health promotion, team work, community participation or research, to name a few examples.(6)

The feeling of lethargy in relation to PHC is the most common perception among professionals who are still active; lethargy that is only saved sporadically by some proposals of rebellion against that established and of proposals suggesting that at the moment they just stay at that. Although the realities differ based on the contexts from the different countries, we believe some common causes to all of them can be established, at least in great part, which justify the reasons why today PHC is still being talked about as an objective to be achieved, when nearly 45 years have passed since Alma Ata.

For such, and based on that indicated by Pérez-Giménez, we identify several key processes:(7) (i) Mimicry of the biomedical, paternalistic, medicalized, and fragmented model of most Public Health Systems that impedes the development of the principal proposals of PHC regarding comprehensive participatory care, universalization, accessibility, equity; (ii) Lack of specific legislation that regulates the organization of PHC and its articulation with national health systems or the effective development of those existing in some countries; (iii) Lack of continuity in the changes begun after the Alma-Ata Declaration, which led to the philosophy of the PHC being talked about on many occasions, placing it on the plane of utopia or desire, but not of the real concretion; (iv) Idealization of Team Work that collides with the struggle of interests from the different professional collectives when interpreting that there is invasion of competences by some professionals, as is the case of nurses and the leadership role they acquire in many settings of PHC; (v) Suspicion of active and direct participation by the community in decision making, which is interpreted by some health professionals as an interference from the paternalistic and autocratic perspective imposed by themselves; and: (vi) Increase in liberalizing measures, whose symbol is the empowerment of new forms of management that do not identify PHC as a business model, leading to poor or meager funding that makes it unfeasible in relation to the objectives set forth in the Declaration and to a progressive reduction of the substantivity of the right to health caused:

This last key process is represented by:

- Distribution and adaptation of resources with clear criteria of inefficiency.
- Important costs increase and budget cuts for primary care. Considerable increase in demand without an adequate response to it.
- Significant increase in medicalization, without improvement in the population's perception of health; progressive incorporation of technology with displacement of promotion and prevention activities.
- Constant and fleeting experiences that fail to respond to the growing needs raised by the community and generate mistrust, demotivation, and rejection in professionals.
- Abandonment of basic PHC principles, such as community participation and intervention; greater lack of coordination between levels of care with the consequential decrease in the continuity of care.
- Bureaucratization of care that makes it more distant and impersonal.
- Underuse of the resolution capacity of professionals, such as nurses or social workers, increasingly dedicated to care and administrative tasks.
- Ineffective organizational models in which the needs of the system or of certain health groups prevail over those of the community.
- Invisibility of care when not institutionally incorporated as a nursing product of its own in PHC.
- Implementation of information technology systems with purely administrative objectives.
- Lack of adequate and continuous formation, as well as specialization by nurses.
- Little or no collaboration with scientific societies.
- Ineffective or non-existent planning of medical and nursing consultations, aimed at responding to the growing demand without criteria of quality, effectiveness, and efficiency.
- Increased pharmaceutical spending and, paradoxically, in therapeutic abandonment.
- Scarce attention to vulnerable groups in the community, such as the frail elderly, the terminally ill, the mentally ill, the chronically ill, etc.
- Increase in individualistic care to the detriment of family and community intervention.
- Little or no team work.
- Organization on the basis of activities and tasks that reduce the autonomous action capacity of professionals.
- Progressive isolation of the health center and its professionals from the community.
- Scarce programmed activity.
- Diminished home care.
- Stagnation or decline in research, derived from the lack of support and general lack of motivation.
- Activity indicators that provide very timely information and with scarce relevance for management.

## PHC and nursing leadership

To face the changes taking place to meet the new demands of the population and to achieve a PHC of the future, the most basic and conceptual aspects of the model continue being appropriate. Particularly, it is appropriate to recognize and enhance in the model the now classic defining attributes of PHC: accessibility (being close to the homes of the people and accessible 24 hours of the day, every day of the year), globality (attending to the different aspects of people's lives), longitudinality (doing so throughout the life cycle of individuals), and integrality (the professional's ability to provide comprehensive, integrated and integrative care to individuals, families and the community in the physical, mental, social and spiritual spheres) and bearing in mind the personal, family, social, and community resources available for its adequate and effective articulation and coordination).(8)

In spite of the validity of these fundamental elements of the model, the orientation of primary care will have to provide satisfactory answers to the new needs for adaptation and improvement of services like those that, for example, are proposed after the pandemic that has left behind a clear context of care that must necessarily be taken care of and led. Special emphasis should be placed on those aspects of the model that are insufficiently developed (for example, community care, as was also evident during the pandemic) and on those other aspects that, from an innovative perspective, to meet the new requirements of quality, equity and efficiency in providing services and satisfying citizens in this respect.(9)

## Changes to improve PHC

Changes that seem reasonable to improve primary care coincide practically with those indicated by Vuori (10) when the PHC reform began as consequence of the Alma-Ata Declaration and which will be explained ahead:

## Conceptual

Primary Health Care must focus, progressively, more on the citizens, their health problems, and the processes it must address than on the structures where care is provided. It does not seem logical to continue building infrastructures and that the actions carried out therein fall within the scope of the medical care that led to the primary care reform. That is, exclusively focused on care of individualized demand, disease, and pharmacological treatment. This leads to a vision of the organization that is completely different from the current stratified pyramidal type.

The improved PHC's resolving power must be identified as an instrument to achieve the most effective and efficient way of positively influencing the individual, family, and community health situation (health indicators) and, through such, the quality, life expectancy, and level of overall satisfaction of the people. It is necessary to identify as objective the improved health level and, through it, the quality of life, both from a population and individual perspective. This implies developing intersectoral strategies in which public health systems and other community resources are involved, reaffirming and strengthening community participation and intervention.(11) It is essential to incorporate the contributions to community health made by different staff professionals. The tendency to forget or underestimate the fundamental influence that these inputs have on the final decision-making power must be eliminated. Another centrally important element is that of assessing the quality and relevance of care actions with a level of priority equal to or higher than that given to their quantity.

## Funding

It is necessary to design new forms of financing in the medium and long term to have a forward-looking vision that will allow us to provide an adequate response to the needs and demands of our clients. Resources should be allocated in a way that does not depend on keeping the structures to which the systems are often subjected. Structures cannot be justified on their own, rather, on the basis of the service they provide.

The payment systems of the different levels of care must be standardized to monitor the final destination of resources and the results obtained in the provision of services. The traditional strength and capacity of influence of the hospital sector within the productive structure of the offer of health services leads to its primacy with respect to the distribution of resources, with the consequent detriment to PHC. A more active policy that would permit the redistribution of resources would lead to a fairer and more balanced internal distribution of the budget that would allow to adequately strengthen PHC as a level of care that makes an important contribution to the systems as a whole. The constraints of a hospital level that acts as a lobby and hinders the necessary changes in the functional distribution of the budget must be limited as soon as possible. Restrictive conditions of the political and economic situations of many countries limit the PHC progress, but should not be a permanent excuse for its present and future paralysis.

## General organizational

Health legislation must be adapted to the evolution of health care models to achieve the necessary organizational changes and unblock paralysis situations caused by the lack of regulatory coverage or even by the opposition of existing health legislation. Primary Health Care cannot be oblivious to the important socio-demographic, economic, political and family changes and to the demands that derive from them. The organization is centered fundamentally on responding to acute problems and to technological progress, while the emerging needs are increasingly focused on the problems associated with chronicity, dependency, social determinants or the Sustainable Development Goals.

To effectively address this reality, cross-sectoral strategies are essential. Community-based health care should promote attitudinal changes and education and training activities, as well as to be applied in all social and health care activities to make them accessible, comprehensive, integrated, inclusive, responsive to people's needs and cost-effective. It is a reality that there are two well-defined subsystems (technological, sporadic care, focused on the disease, which takes place in hospitals, and continuous and continued personal, family, and community care, centered on the primary care/social-health care conglomerate), which increasingly evidence the need to generate links that coordinate and integrate them through their priority inclusion in service contracts (symmetrically and complementarily in both primary care and hospital care contracts).

It is necessary to transform the coordination strategies between professionals and levels for others of vertical and horizontal integration of care by implementing specific strategies and evaluating their degree of compliance in practice to avoid, as has been happening, their remaining as mere declarations of good intentions.

## Management and offer of services

Optimization of equity, effectiveness, and quality of resource distribution and health actions must be the consequence of balanced health resource management. Supply of care must achieve equity and distributive justice, providing more to those who need it most and have the least possibilities of solving their own problems. Therefore, the supply of (public) health services must always prioritize vulnerable population groups, offering basic public health services complemented by a broad offer that meets the needs of the most disadvantaged, without falling into charity services that increase the differences between health care provided to the rich (private) and the poor (public). The provision of aspects of the PHC service portfolio underdeveloped in the care offered by the staff (group care activities, pediatric home care, school health, home care, among others) should be included and strengthened.

Rapid aging of the population poses a huge challenge to PHC providers, and the demand for said services may be increased as the number of elderly adults increases in the population. The global disease profile is changing. A clear transition exists from infectious diseases to noncommunicable and chronic diseases, most of which can be prevented or delayed through health promotion and disease prevention strategies. Constant monitoring of chronic diseases is required to minimize the occurrence of associated disabilities and their adverse effects on quality of life, along with a strong PHC that can respond to emergency situations, such as COVID-19 or other possible pandemics in coordination with Public Health Services from a global perspective.(12)

Lack of qualitative methodologies and insufficient impact of PHC evaluation results contradict, on the one hand, the general conviction about the importance of service evaluation and, on the other hand, the lack of a clear and comprehensive evaluation methodology, and moreover, with the fact of having opted for a more-flexible model from the normative point of view, regarding the reference to the structure and care process, and in that the control of services centers theoretically on obtaining certain health results and citizen satisfaction.

## Local organizational aspects (of daily work)

Allocation of resources based on population criteria and adjusted to real needs, fundamentally care needs, must contribute to rationalizing the staffing of PHC professionals with a significant increase in the number of general-care community nurses and the progressive incorporation of nurses specialized in family and community nursing who are in the best conditions and capacity to lead these care processes, especially after the pandemic (secondary effects to persistent COVID or as a consequence of the lack of attention during the pandemic to certain problems, like chronicity, palliative care, family caregivers, gender violence, mental health, as examples).(13)

Despite significant organizational changes produced in PHC, more as a mimetic response in its beginnings to the novelty derived from the Alma-Ata Declaration than to a real identification and will for it, these not only have not evolved to adapt to the needs of the community, but rather there has been a regression towards organizational approaches prior to the aforementioned changes. Another noteworthy fact is the poor distribution of skills in care actions that persists among the groups of professionals and that does not follow criteria to optimize effectiveness and efficiency. It is essential to perform a serious reflection and in-depth analysis on this issue that allows reorienting the priority fields of professional action, with special reference to community nurses and family doctors, with emphasis on the essential transdisciplinary work capable of overcoming problems derived from competence barriers by identifying common care objectives above the corporate and corporatist objectives of the different disciplines.

Teaching activities of the staff professionals must be extended to undergraduate and graduate studies in all the professional categories of the staff and facilitate accreditation of services for the teaching exercise. It is necessary to recognize in the contracts with provider entities and during the working hours of care professionals the time slots allocated to teaching and research activities and, particularly, promote support and labor recognition of the tutorial action of care professionals.

## Professional

Professionals must keep in mind the needs, expectations, and demands of citizens to provide effective and efficient responses removed from the individualistic perspective that as collectives they often set as priority and which provoke not only the exclusion of that perspective, but also the inefficiency of the system as a whole. Priority should be assigned to those aspects of the care offer most directly related with caring for people, especially with respect to their expectations, level of demand regarding health services, and development of the relation of trust between the PHC professional and the individual (time accessibility, treatment personalization, confidentiality, dedication time, quick response, humanization, active listening, etc.).

The PHC professionals must come together in a true culture of transdisciplinary teams that reduces ineffectiveness and inefficiency in care processes derived from their absence. Nurses must be incorporated as true protagonists in improving the resolution power of PHC, specifically in home and community care actions. Likewise, it is necessary to tend to give prominence to professionals through the promotion of decision-making capacity, enhancing their motivation and real initiative capacity. Professionals must include the scientific evidence available in their management and professional-practice decisions, which favors abandoning the defensive practice often generated by mistrust between managers and professionals.

## Users

Citizens must be well informed and acquire a responsible culture of resource use that allows them, in turn, to demand prompt and quality care, so that they can influence the resolution power of the PHC. It is essential to promote and strengthen the positive image of PHC, moving it away from the conception, still present in many politicians, managers, professionals and citizens, that it is a minor or second-class service compared with the resolution preponderance of technology and scientific prestige of hospitals. Confidence in PHC will favor its decision-making power, avoiding unsatisfied demand, dependency, deviation of care demands, and imbalance of budget allocations.

## Participation

The perspective of improving PHC must not be limited to the clinical resolutive power. Community and non-medical resource settings must be purposefully and firmly incorporated, given that these elements also define a significant part of the quality of care. Community, citizenship, and organization participation in all the phases of the planning, management, and evaluation processes must be constituted as a key element in the future perspective of PHC, through effective mechanisms. This participation must avoid rigid and bureaucratic positions to achieve the necessary and desirable flexibility and adaptation to the characteristics of each environment.(14)

## Future perspective of primary care

In spite of the deficiencies and deterioration of PHC, the necessary changes have not taken place to reverse the situation, which has been clearly evidenced during the COVID-19 pandemic.(15) Said deterioration has become more profound as a consequence of the austerity policies rightfully carried out or justified by crises and by demographic, social, family structure, and epidemiological changes, among others. The debate on the need to adapt the current PHC model that responds to the needs of the population is undergoing a paradigm shift in which individuals, families and the community are the true protagonists and health professionals from other areas and community health agents participate in coordinated and planned manner in addressing health problems, needs, experiences, and expectations of the people according with their bio-psychosocial and spiritual context, going beyond the disease and focusing on health through a salutogenic approach based on health assets and having health promotion as a transversal axis and with intersectoral approaches and transdisciplinary work.

To achieve this, nurses are needed. Not because it is nurses who will respond exclusively to the needs that arise from the PHC, but because nurses are in the best position and have the knowledge and skills to effectively and efficiently address this challenge. Nurses are not only used to join health institutions. It is necessary to identify the importance of incorporating nurses who will lead this process and who manage to coordinate resources and structure actions from the participatory work of all health agents, whether professionals or not, whether they are part of health or not. What is needed is professional nursing care. Permanent and fluid communication among the different community resources (both public and private) may also be necessary, and especially with universities to design, adapt, and implement specific skills in future nurses that allow them to be trained to deliver the best responses to situations, such as the one we are dealing with. The care that must be provided to future prospects must also be contemplated, promoted and prevented.

Care, on the other hand, is not exclusive to nurses, only professional nursing care is. But, undoubtedly, those who know best how to identify, assess, and manage care are nurses in general and community nurses in particular.

It is important to lay the foundations that allow contextualizing the scenario in which you want or seek to intervene to, from there, start agreeing on actions; in the same way that it is essential for nurses to lead many of these processes.

To conclude and as a summary, I present what I consider are the key words/ideas of the reformulation of PHC:

Care context, comprehensive care, integrated and integrating, health assets, healthy environments, community resources, inter-sectoriality, trans-disciplinarity, respect, generosity, gratitude, vulnerability, health literacy, community participation, and of course - NURSES.
